# Novel Myco-Coagulant Produced by *Lentinus squarrosulus* for Removal of Water Turbidity: Fungal Identification and Flocculant Characterization

**DOI:** 10.3390/jof8020192

**Published:** 2022-02-16

**Authors:** Nessa Jebun, Md Zahangir Alam, Abdullah Al Mamun, Raha Ahmad Raus

**Affiliations:** 1 Department of Biology, Presidency International School, Chattogram 4217, Bangladesh; jabucu2005@gmail.com; 2 Bioenvironmental Engineering Research Centre (BERC), Department of Biotechnology Engineering, Faculty of Engineering, International Islamic University Malaysia (IIUM), Gombak, Kuala Lumpur 50728, Malaysia; 3 Bioenvironmental Engineering Research Centre (BERC), Department of Civil Engineering, Faculty of Engineering, International Islamic University Malaysia (IIUM), Gombak, Kuala Lumpur 50728, Malaysia; mamun@iium.edu.my; 4 Bioprocess and Molecular Engineering Research Unit (BPMERU), Department of Biotechnology Engineering, Faculty of Engineering, International Islamic University Malaysia (IIUM), Gombak, Kuala Lumpur 50728, Malaysia; rahaar@iium.edu.my

**Keywords:** *Lentinus squarrosulus*, myco-coagulant, flocculation mechanism, turbidity, water treatment

## Abstract

Several river water fungal strains (RWF-1 to RWF-6) were isolated to investigate the potential of having coagulant properties from the metabolites produced by the fungus. The myco-coagulant produced from the liquid-state process was characterized and tested for flocculation of kaolin water. Molecular identification of the fungal strain isolated from river water and characterization of the myco-coagulant produced by the strain are presented in this paper. The genomic DNA of the fungal 18S ribosomal ribonucleic-acid (rRNA) and 28S rRNA genes were used and the species was identified as *Lentinus squarrosulus* strain 7-4-2 RWF-5. The characterization of myco-coagulant by Fourier-transform infrared spectroscopy (FTIR) showed that hydroxyl, carbonyl, amide and amine groups as principal functional groups were present in the new myco-coagulant. The mean zeta potential value of the myco-coagulant was −7.0 mV while the kaolin solution was −25.2 mV. Chemical analyses of the extracellular myco-coagulant revealed that it contained total sugar (5.17 g/L), total carbohydrate (237 mg/L), protein (295.4 mg/L), glucosamine (1.152 mg/L); and exhibited cellulase activity (20 units/L) and laccase activity (6.22 units/L). Elemental analyses of C, H, O, N and S showed that the weight fractions of each element in the myco-coagulant was 40.9, 6.0, 49.8, 1.7 and 1.4%, respectively. The myco-coagulant showed 97% flocculation activity at a dose of 1.8 mg/L, indicating good flocculation performance compared to that of polyaluminum chloride (PAC). The present work revealed that the fungal strain, *L. squarrosulus* 7-4-2 RWF-5 is able to produce cationic bio-coagulant. The flocculation mechanism of the novel myco-coagulant was a combination of polymer bridging and charge neutralization.

## 1. Introduction

Coagulants are used in various activities, including water purification, wastewater treatment, food preparation, medication industries and fermentation. Industrial downstream processes also require various types of coagulants [1,2]. Generally, chemical coagulants are used in water treatment facilities due to their rapid production in bulk amounts at affordable costs [3,4]. However, the excessive use of these chemically produced coagulants may lead to various issues [5]. Aluminium content in poly aluminium chloride (PAC), which is a common coagulant used in the water industry, is associated with Alzheimer’s disease [6]. In order to combat the health risks due to aluminium-based coagulants, research works have been on-going to develop safe, environmentally friendly and degradable coagulants. On the other hand, acrylamide monomer residues in polyacrylamide are carcinogenic and neurotoxic to human beings [7].

There are many drawbacks of chemical coagulants and it is very essential to explore eco-friendly and safe bio-coagulants which could be an alternative to chemical coagulants. Compared to the chemical coagulants, bio-based coagulants have greater advantages in being eco-friendly due to greater biodegradability, less pollution potential and less threat to the ecosystems. Microbial secondary metabolites are usually made of proteins, glycoproteins, polysaccharides and lipids, which are produced during the growth time and can act as a bio-coagulant [8]. The extracellular polymeric substances secreted by microorganisms can be used to reduce turbidity. Several researchers have reported that the bio-flocculation mechanism is responsible due to polysaccharides and proteins which contribute to the positive charge of bio-coagulant [9]. For example, protein from *Bacillus mucilaginosus* [10,11], a glycoprotein from *Bacillus sp.* [12], protein and polysaccharide [13,14], sugar and protein [15,16] and carbohydrate and protein from *Bacillus salmalaya* strain 139SI [17] have been reported as various forms of bio-coagulants for water treatment.

The novel bio-coagulant produced by *Aspergillus niger* due to the presence of Ca^2+^ ions had ability to remove 63% of turbidity and to purify river water from colloids [18,19]. The presence of CaCl_2_ formed by *Bacillus licheniformis X14* was found to increase the removal of turbidity from water. Moreover, *Bacillus sp.* could reduce turbidity from kaolin suspension and Tyume River water at 85.8% and 86.9%, respectively [12]. Similar results have been found for *Serratia ficaria*, which showed 84.2% turbidity removal from Xiaoqing River with the addition of CaCl_2_ [20]. *Azotobacter chrococcum* showed 81% turbidity removal in the presence of CaCl_2_ from river water [21]. Consequently, the flocculation process with the addition of cations can produce secondary pollution and increase costs. Therefore, it is necessary to find bio-coagulants which can be a potential resource without the addition of any cationic coagulants.

In this study, a novel myco-coagulant produced by *Lentinus squarrosulus* strain 7-4-2 RWF-5 showed high coagulation capability from kaolin suspension and river water without adding cations. This paper reports the identification of fungal strain and characterization of the novel myco-coagulant. Physio-chemical properties of the myco-coagulant and its flocculation behaviour are reported too.

## 2. Materials and Methods

### 2.1. Sample Collection

The fungi were isolated from river water sample using spread plate technique. The water sample was collected from the Pusu River, which is flowing through IIUM Gombak Campus [22,23]. The samples were inoculated onto the PDA plates prepared in the laboratory. The inoculated plates were kept for 5–7 days at room temperature (30 ± 2 °C). Pure cultures were obtained aseptically by picking up new fungal colony and transferring into fresh sterile PDA plates.

### 2.2. Morphological and Molecular Identification

The fungal morphology was investigated based on external growth and visualization through a microscope attached to a digital camera (National DC5-163 Digital, National Optical & Scientific Instruments, Inc. Schertz, SA). For molecular identification, a DNA sample was collected from the pure fungal strains grown for 10 days. Genomic DNA extraction, PCR amplification, purification of the PCR products and the determination of sequences were performed by an accredited laboratory named First BASE Laboratories Sdn. Bhd. (Company Reg. No. 604944-X, Selangor, Malaysia). For molecular identification of the strain, DNA was extracted and then the fractionated DNA bands were obtained and compared with a known DNA molecular marker (*Flammulina velutipes*) by using agarose gel electrophoresis. The forward and reverse sequencing results were assembled into one complete sequence then compared with the top 10 hit blast results against NCBI Nucleotide Collection (nr/nt) database. After that, a phylogenetic tree was drawn using BLAST pairwise alignments and the neighbour-joining method [24].

### 2.3. Production of Myco-Coagulant

The best medium for increasing myco-coagulant production by *L. squarrosulus* RWF-5 was investigated. After mixing the malt extract with 1 litre of distilled water in a ratio of 0.1% (*w*/*v*), the media was sterilized by autoclaving it at 121 °C for 15 min, followed by inoculation with 3% (*v*/*v*) fungal strength (dry mycelia 340 mg/L) [22]. Ten days old culture plates were used to prepare fungal inoculum, which was incubated for 6 days in a rotary shaker at 150 rpm (at 30 ± 2 °C). The initial pH of the culture was adjusted to 7.0 ± 0.1 by using 1M NaOH or 2M HCl. The culture, together with the media, was harvested after six (6) days of cultivation, to extract the potential myco-coagulant.

### 2.4. Separation and Purification of Myco-Coagulant

The separation of myco-coagulant was carried out by centrifuging the broth at 10,000 rpm (at 25 °C) for 10 min. Then the supernatant was collected to be used as a myco-coagulant. To precipitate insoluble substances, two volumes (2:1) of cold ethanol (4 °C) was added into the supernatant after 10 min to indicate precipitation in the upper phase of the mixture. The precipitates were collected and washed with deionized water, followed by a drying process using a vacuum system to get concentrated myco-coagulant [16].

### 2.5. Characterization of the Myco-Coagulant

Surface morphology and zeta potential: The surface morphology of purified dry myco-coagulant, kaolin particles and coagulant with flocculated kaolin were elucidated using Field Scanning Electron Microscopy- FSEM (JEOL, JSM-6700F, Peabody, MA, USA). The zeta potential was measured for kaolin suspension (0.7 g/L), control (0.1% *w/v* malt extract broth) and 2, 4, 6 days old supernatants using a Zetasizer Nano ZS at 25 °C.

Chemical analysis: The total carbohydrate and sugar content of the myco-coagulant were determined using the phenol-sulfuric acid method, with glucose as the standard solution [25]. The Folin phenol reagent method was used to determine total protein content, with bovine serum albumin as the standard [26]. Chitin estimation (using glucosamine as standard) was done according to the method of Chen and Johnson [26]. The cellulase enzyme assay was determined using the filter paper method [27]. Laccase activity assay was determined by the method which is based on the oxidation of ABTS [28]. Elemental analysis was done for CHNS/O by Perkin Elmer through elemental analyser (Perkin Elmer Series II, Long Island, NY, USA). The functional groups of the bio-coagulant were determined using a FTIR-Spectrum 400 of Perkin Elmer, USA.

Flocculation performance of myco-coagulant: The flocculation efficiency of the myco-coagulant and polyaluminium chloride (PAC) were determined using kaolin suspension. Synthetic turbid water was prepared using 0.5 g kaolin powder (R & M chemicals, UK) in 1 L of distilled water (turbidity 600 ± 10 NTU). Different dosages of myco-coagulant (%*v*/*v*) were added into every jar, which contained 500 mL kaolin suspension with known turbidity. To investigate the synergetic effect of cations on the flocculating activity of myco-coagulant, various cations were used which included Al_2_ (SO_4_)_3_, FeSO_4_, NH_4_Cl, NaCl, KCl, MgCl_2_, CaCl_2_ and FeCl_3_. The effect of pH on the flocculating activity of myco-coagulant was investigated by adjusting pH values from 4 to 8, using 2MHCl or 1M NaOH (depending on the value of pH). The jar apparatus (Stuart, Flocculator SW6, UK) was rapidly stirred at 250 rpm for 7 min, slowly stirred at 90 rpm for 28 min, and then allowed to settle for 5 min [22]. The supernatant from every jar was collected for residual turbidity measurement (Turbidimeter 2100Q, HACH, Loveland, CO, USA). Flocculation activity was calculated using Equation (1) [29].

(1) $$\mathrm{Flocculating}\;\mathrm{activity}\;\left(\%\right)=\left[\frac{A-B}{A}\right]\times 100\%$$

where, *A* = initial turbidity (NTU) and *B* = residual turbidity (NTU) after flocculation.

## 3. Results and Discussion

### 3.1. Identification of Fungal Strain

**Figure 1:**
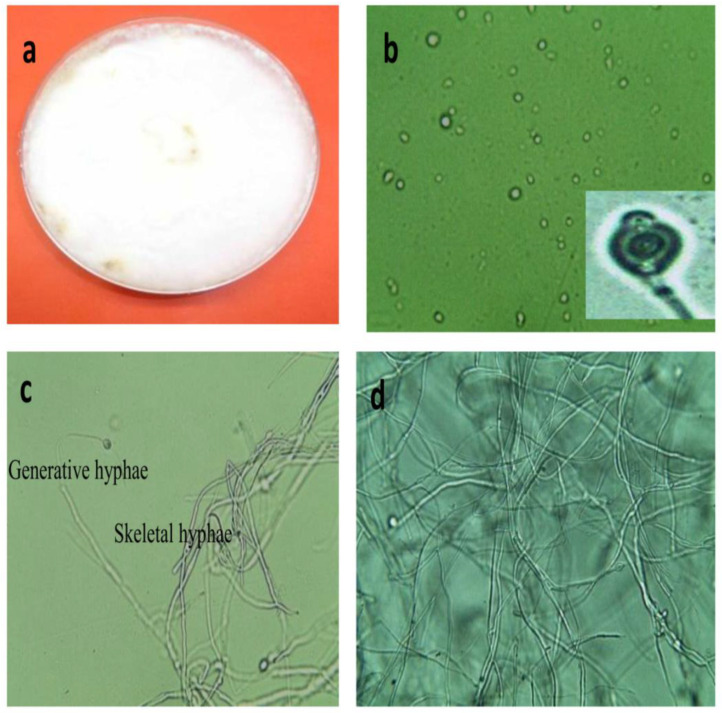
Morphology of *Lentinus squarrosulus* after 10 days. (**a**) On PDA plates, (**b**) fungal basidiospore, (**c**,**d**) hyphal structure at 400× magnification.

Six filamentous fungi (RWF-1 to RWF-6) were isolated and screened based on their ability to in terms of reduce turbidity from river water samples [22]. The best performing fungus that produced good quality myco-coagulant was identified as *Lentinus* genus. This finding was based on fungal-colony morphology on PDA (Figure 1a). Microscopic images of the basidiospores and fungal hyphae are shown in Figure 1b–d.

Based on the presence of dimitic and amphimitic hyphal systems, *Lentinus* has been grouped under the family *Polyporaceae* [30,31]. Microscopic images of hyphae and basidiospore results are in agreement with the findings of [32] which described the generative and skeletal hyphae, basidiospore shapes were ellipsoid. In the beginning, the new fruiting bodies were whitish in appearance. They turned brownish at the maturity stage. These morphological studies showed that this identified strain was under the *Lentinus* genus.

**Figure 2:**
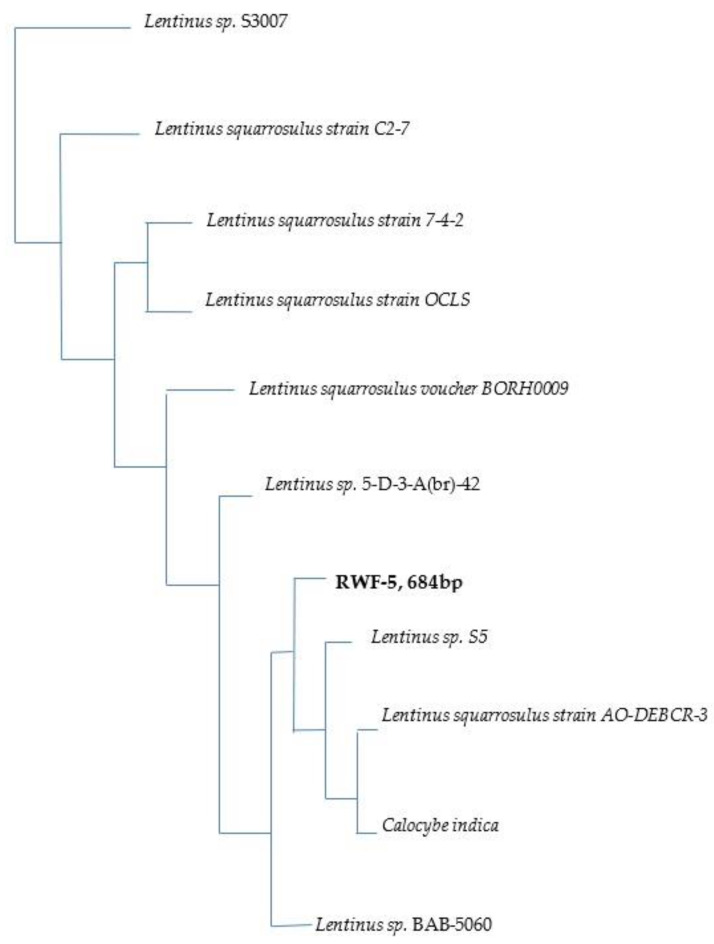
Phylogenetic tree based on Tree Neighbour Joining (Unrooted Tree) Method. (Detailed description: *Lentinus sp.* S3007: 18S ribosomal RNA gene, partial sequence; internal transcribed spacer 1, 5.8S ribosomal RNA gene, and internal transcribed spacer 2, complete sequence; and 28S ribosomal RNA g; *Lentinus squarrosulus* strain C2-7: 18S ribosomal RNA gene, partial sequence; and internal transcribed spacer 1, 5.8S ribosomal RNA gene, internal transcribed spacer 2, and 28S ribosomal RNA gene; *Lentinus squarrosulus* strain 7-4-2: 18S ribosomal RNA gene, partial sequence; internal transcribed spacer 1, 5.8S ribosomal RNA gene, and internal transcribed spacer 2, complete sequence; and 28S ribosome; *Lentinus squarrosulus* strain OCLS: internal transcribed spacer 1, partial sequence; 5.8S ribosomal RNA gene and internal transcribed spacer 2, complete sequence; and 28S ribosomal RNA gene, partial; *Lentinus squarrosulus* voucher BORH0009: 18S ribosomal RNA gene, partial sequence; internal transcribed spacer 1, 5.8S ribosomal RNA gene, and internal transcribed spacer 2, complete sequence; *Lentinus sp.* 5-D-3-A(br)-42: 18S ribosomal RNA gene, partial sequence; internal transcribed spacer 1, 5.8S ribosomal RNA gene, and internal transcribed spacer 2, complete sequence; and 28S ribosome; *Lentinus sp.* S5: 18S ribosomal RNA gene, partial sequence; internal transcribed spacer 1, 5.8S ribosomal RNA gene, and internal transcribed spacer 2, complete sequence; and 28S ribosomal RNA; *Lentinus squarrosulus* strain AO-DEBCR-3 internal transcribed spacer 1, partial sequence; 5.8S ribosomal RNA gene, complete sequence; and internal transcribed spacer 2, partial sequence; *Calocybe indica* internal transcribed spacer 1, partial sequence; 5.8S ribosomal RNA gene and internal transcribed spacer 2, complete sequence; and 28S ribosomal RNA gene, partial; *Lentinus sp.* BAB-5060 18S ribosomal RNA gene, partial sequence; internal transcribed spacer 1, 5.8S ribosomal RNA gene, and internal transcribed spacer 2, complete sequence; and 28S ribosomal R.)

Molecular identification of *Lentinus sp.* was most closely identical to the fungal strains based on rRNA operon gene sequences. Gene sequences from 10 *Lentinus sp.*-related strains were compared to the sequence-similarity studies of fungal rRNA operon in GenBank to see how they were similar. Referring to the nucleotide sequence, the filamentous strain was 99% similar to the *Lentinus squarrosulus* type strain 7-4-2 (GenBank accession Number GU001951.1). As such, the RWF-5 was identified as *Lentinus squarrosulus* by both its morphological and phylogenetic characteristics. Gene sequences of the 10 strains with maximum sequence identity and nucleotide sequences (bp) difference were used to draw a phylogenetic tree. A phylogenetic tree was constructed between it and similar sequences were found in GenBank (Figure 2). This tree was produced by NCBI Blast Tree Neighbour Joining (Unrooted Tree) Method.

### 3.2. Characterization of the Myco-Coagulant

Determination of Zeta potential: The average zeta potential value of the supernatant (myco-coagulant) after 6 days of cultivation of *Lentinus squarrosulus* strain 7-4-2 RWF-5 was −7.01 mV, while values for the control and kaolin were −26.5 mV and −25.2 mV, respectively. The zeta potential values of 2- and 4-day-old myco-coagulant were −20.36 mV and −14.2mV, respectively.

The results indicated that the myco-coagulant released positively charged particles that have reduced the zeta potential values compared to those of 2- and 4-day supernatants. The low zeta potential indicated that charge neutralisation and good flocculation had occurred. Subramanian et al. [9] also reported analogous result, which revealed that *Bacillus*-derived extracellular polymeric substance (EPS) had lesser negative charge than fresh sludge. With and without the addition of Ca^++^, EPS performed better in kaolin suspension in actual sludge settling. According to the results, charge neutralization was also found to be the primary mechanism of the flocculation process. This is because the myco-coagulant is a positively charged bio-coagulant which is independent of cations.

**Figure 3:**
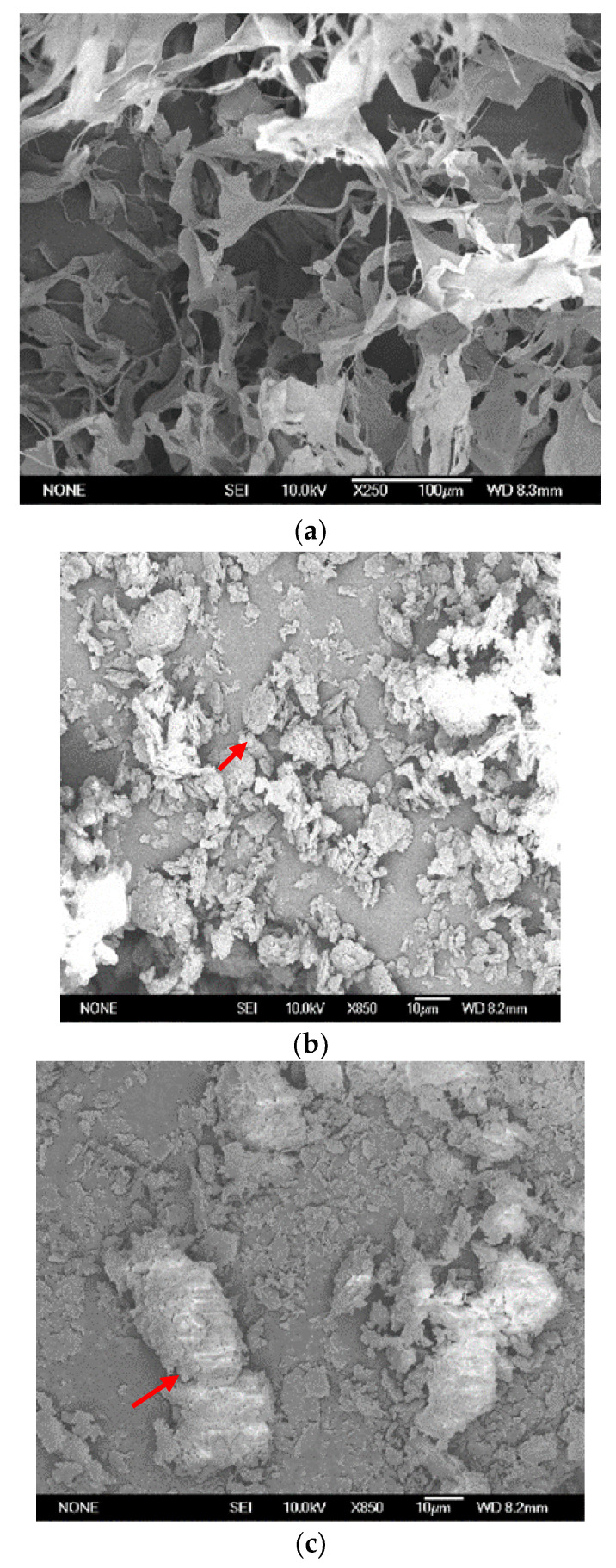
Scanning electron microscopy (SEM) images. (**a**) Purified myco-coagulant, (**b**) kaolin particles before flocculation, (**c**) kaolin clay flocculated by myco-coagulant (Arrows show small particle in kaolin solution alone and bigger particles after flocculated by myco-coagulant).

Surface structure analysis: The supernatant yielded 0.20 g of pure RWF-5 fungus from 1.0 L of culture media. The freeze-dried purified myco-coagulant was found to be white in colour. Morphology of separated myco-coagulant and scattered kaolin particles before flocculation are shown in the SEM images in Figure 3a,b. When the myco-coagulant was used, the kaolin particles flocculated into larger flocs, as shown in Figure 3c. According to Alam and Razi [33], a mixed culture *of Aspergillus niger* and *Penicillium corylophilum* strains immobilized/entrapped sludge particles by producing pellets that improved the separation process.

Pure myco-coagulant had an irregular, coarse-grained structure linked in a netted pattern. The myco-coagulant molecule’s structure may play a role in its high flocculating efficiency [34]. SEM pictures of the myco-coagulant and the flocculated kaolin clay demonstrated that polymer bridging also can be another mechanism of the flocculation process of this new myco-coagulant discovered in this research work. Similar finding was reported for bio-coagulant produced from *Bacillus licheniformis* strain W7 [12]. It was understood that ionic forces would bring the coagulant molecules and kaolin particles close enough to form flocs throughout the flocculation process. A bridging mechanism is reported to help adsorb the particles onto the bio-coagulant’s chain of molecules [19].

### 3.3. Chemical Analysis

Composition of myco-coagulant: Chemical analyses of the extracellular myco-coagulant consisted of total sugar of 5.17 (g/L), total carbohydrate of 237 (mg/L), protein of 295.4 (mg/L), glucosamine of 1.152 (mg/L), cellulase activity of 20 (units/L) and laccase activity of 6.22 (units/L).

The edible mushroom *Lentinus squarrosulus* RWF-5 is generally found in the woods and on dead trees. Proteins, sugars, lipids, amino acids, vitamins and minerals are abundant in the fruit’s flesh [35]. The reason behind the decomposition of lignin by white-rot fungi is the extracellular enzyme system secretion. Several reports [36,37] showed that laccases are glycoproteins with molecular weight (MW) of roughly 70 k Da derived from white-rot mushrooms. This study revealed that there is a strong possibility that the active compound of myco-coagulant is a biopolymer containing carbohydrates, proteins and glycoproteins as the primary components. The elemental level composition of the dried myco-coagulant revealed that it consists of 41.9% carbon, 6.0% hydrogen, 49.8% oxygen, 1.7% nitrogen and 1.4% sulphur.

**Figure 4:**
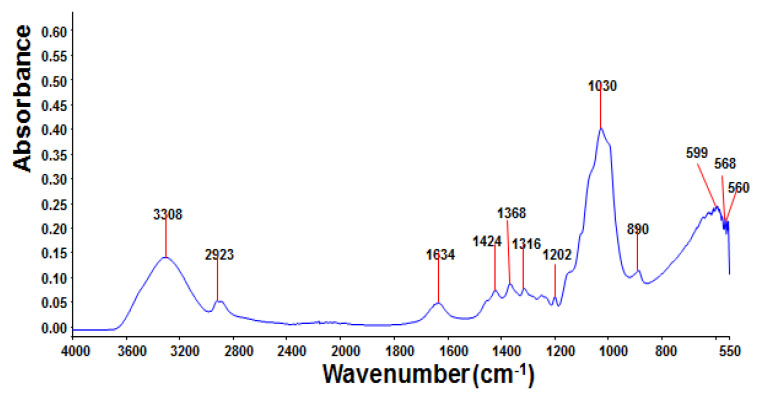
FTIR spectra of purified myco-coagulant produced by *Lentinus squarrosulus* RWF-5.

Functional group analysis: The purified myco-coagulant was subjected to FTIR spectroscopy (Figure 4). Pure myco-coagulant has a wide-stretching peak at 3308 cm^−1^ that might be linked to hydroxyl and amine groups in the spectrum, as shown in the FTIR spectrum. An asymmetrical stretching vibration of C-O-H was identified at the peak at 2922 cm^−1^ [12]. The C=O symmetrical stretching in the carboxylate might be assigned to the weak absorption band at 1424, which indicated the presence of a carboxyl group [19]. An asymmetrical broad stretching vibration of a C-O-C ester linkage was observed in a strong absorption band at 1030 cm^−1^, this is a characteristic of all sugar derivatives. The C–H stretching vibration at 890 cm^−1^ was assigned at the peak and a weak band stretching at 568–599 cm^−1^ could be related to the presence of an alkyl halide group.

To further analyse the myco-coagulant, infrared spectra were taken after flocculation of kaolin. The main functional groups present were hydroxyl, amide, amine and ester linkage. These findings show similarities with the FTIR analysis and further proved that OH, N-H, C=O and COC groups were present in the myco-coagulant. It was reported in the literature that in order to enhance the flocculation process, several functional groups such as amine, hydroxyl, carboxyl and ester were the preferred over other groups [12,14,34].

### 3.4. Flocculation Performance

**Figure 5:**
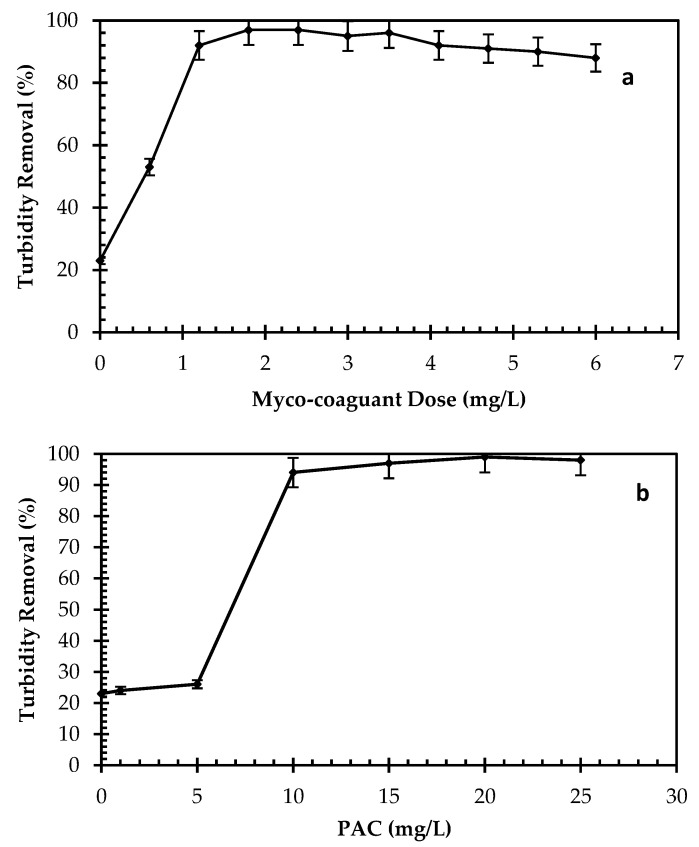
Effects on flocculation performance of (**a**) myco-coagulant, (**b**) PAC (initial turbidity-600NTU, 250 rpm for 7 min 90 rpm for 22 min, pH 7.0, settling time—5 min).

Effect of myco-coagulant concentration: Figure 5a demonstrates the optimum myco-coagulant dose to reduce turbidity from kaolin suspensions. The flocculation rate of the myco-coagulant was studied with dosages varied @ of 0, 2, 4, 6, 8, 10, 12, 14, 16, 18 and 20 mmol/L. The highest turbidity removal of 97% was recorded at 6 mL/L of myco-coagulant in optimized flocculation conditions [38]. These doses had a corresponding protein concentration of 0, 0.6, 1.2, 1.8, 2.4, 3.0, 3.5, 4.1, 4.7, 5.3 and 6 mg/L myco-coagulant, respectively. In comparison, the flocculation rate of Poly Aluminium Chloride (PAC), a common chemical coagulant, was found to be between 0 and 25 mg/L when the identical conditions were used (Figure 5b). The highest flocculation activity could be obtained at PAC dose of 20 mg/L; while only 1.8 mg/L concentration of this new myco-coagulant showed the optimum removal of turbidity. However, a lower PAC dosage showed low flocculation activity to settle the suspended kaolin particles.

The results showed a correlation between high and low dosages of myco-coagulant. It was seen that excessive doses caused the destabilization of the colloids, whereas low doses were insufficient to bridge between biomolecules and colloid particles in the kaolin suspension [12,39].

**Figure 6:**
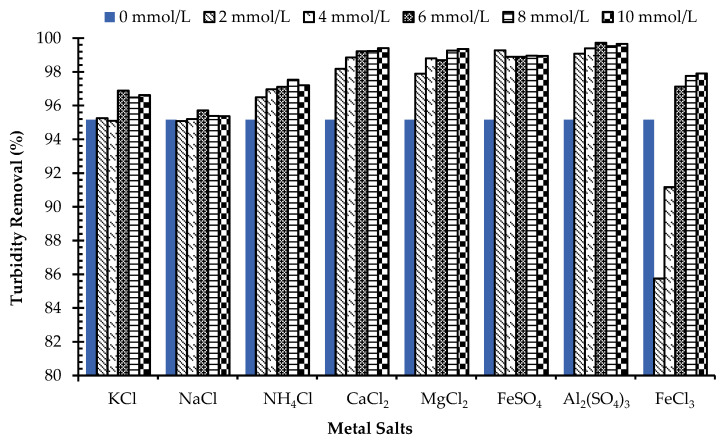
Effects of using metal ions on the flocculation performance of *L. squarrosulus* 7-4-2 RWF-5. (Initial turbidity: 650NTU, pH: 7.0, myco-coagulant dose: 1.8 mg/L, rapid mixing: 250 rpm for 7 min; slow mixing: 90 rpm for 22 min, settling time 5 min.)

Effects of metal ions: Addition of high-valence metal ions during the flocculation process, play a significant role to enhance the destabilization of the colloids in kaolin suspension [12,19,40]. Figure 6 illustrates the flocculating activity of myco-coagulant in kaolin suspension in the presence of monovalent, divalent and trivalent cations. It was observed that monovalent cations Na^+^, NH_4_^+^ and K^+^ (as coagulant aid) have less effect on the flocculation activity of myco-coagulant. Result showed that at pH 7 the myco-coagulant produced 95% of flocculating rate without addition of any metal ions. However, the flocculation activity of myco-coagulant was seen to be slightly stimulated by adding divalent and trivalent cations for, e.g., Mg^2+^, Fe^2+^, Ca^2+^ and Al^3+^, apart for Fe^3+^. The maximum flocculation rate of 99% was achieved with different concentrations of Al^3+^.

The myco-coagulant exhibited promising performance in settling kaolin particles in water, without addition of the cations. Thus, the new myco-coagulant can be considered as a green bio-coagulant because of no generation of secondary pollution due to the presence of any coagulant aid, which is commonly required in water treatment process. Many cation-dependent bio-coagulant has been reported, which produce secondary pollution to the environment such as *Bacillus sp*. [12], *A. niger* [18], and *Chryseobacterium daeguense* W6 [41].

**Figure 7:**
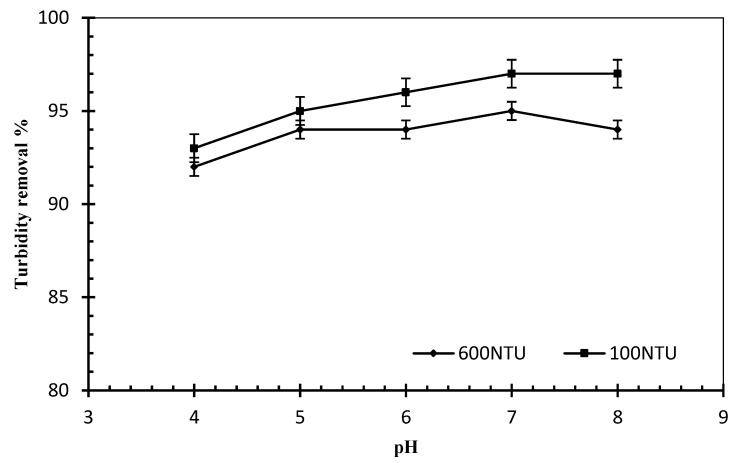
Effects of pH on flocculation performance of *L. squarrosulus* 7-4-2 RWF-5 in different turbid water (myco-coagulant dose concentration-1.8 mg/L, rapid mixing 250 rpm for 7 min; slow mixing 90 rpm for 22 min, settling time: 5 min.)

Effect of pH: The influence of pH on the flocculation process for different turbidity of kaolin is shown in Figure 7. It can be observed that myco-coagulant recorded over 95% of flocculation rate in a wide range of pH at 4 to 8 in high turbid water (initial turbidity 1000 NTU to residual 31 NTU).

In the polymer compound of myco-coagulant, pH-dependent ionization of functional groups displayed full ionization at acidic and alkaline conditions. This resulted in various electric states for the myco-coagulant at varied pH values, which in turn impacted the flocculating efficacy of myco-coagulant on kaolin particles [42]. On the other hand, turbid water (600 NTU) showed high flocculation at pH within 7 and 8. According to the results, the [H+] and [OH] adsorbed under both neutral and alkaline conditions increased the stability of the compound formed between myco-coagulant and kaolin particles. It is reported that various types of bio-coagulants produced by other organisms such as *Streptomyces griseus* [43], *Bacillus sp.* [12] and *A. niger* [39] also worked well in acidic, alkaline and neutral conditions.

The main objective of this research was to evaluate the feasibility of myco-coagulant in removing turbidity from river water. In order for better understanding of the cause and effect of the myco-coagulant, various experiments and laboratory tests were conducted, as highlighted in the previous sections. With successful completion of the initial stage of the study, the team has planned to scale up using solid-state fermentation for mass-scale production of myco-coagulant. Based on the current data, a preliminary economic analysis was done to compare the cost of water treatment using PAC and the new myco-coagulant. One kilogram (kg) of PAC is generally required to treat 100 m^3^ of raw river water; while the myco-coagulant would be required 30 g only. It is expected the cost can be further reduced through optimization of a large-scale solid-state process.

## 4. Conclusions

A novel strain, isolated from river water which is identified as *Lentinus squarrosulus* 7-4-2 RWF-5, produced myco-coagulant. According to the chemical analysis, polysaccharide was found to be the primary component of the glycoprotein. The myco-coagulant was highly potential compared to the conventional chemical coagulant, such as PAC. Flocculation ability of the newly discovered myco-coagulant is tested to be excellent even without any addition of cations, indicating that the myco-coagulant is positively charged. The myco-coagulant showed excellent flocculation activity at wide pH range for highly turbid water (600 to 1000 NTU). It is anticipated that charge neutralization and bridging mechanisms might be involved in the flocculation process of the myco-coagulant.

## Data Availability

Generated during study.
